# Challenges and prospects of snake antivenom supply in sub-Saharan Africa

**DOI:** 10.1371/journal.pntd.0008374

**Published:** 2020-08-20

**Authors:** Abdulrazaq G. Habib, Baba M. Musa, Garba Iliyasu, Muhammad Hamza, Andreas Kuznik, Jean-Philippe Chippaux

**Affiliations:** 1 Health Economics & Outcomes Research (H-CORE) Group, Department of Medicine, Bayero University, Kano, Nigeria; 2 Venom and Antivenom Research Project (VASP), Bayero University, Kano, Nigeria; 3 African Snakebite Research Group (ASRG) Project, Bayero University, Kano, Nigeria; 4 Africa Center of Excellence in Population Health and Policy, Bayero University, Kano, Nigeria; 5 Regeneron Pharmaceuticals, Tarrytown, New York, United States of America; 6 Université de Paris, MERIT, IRD, France; 7 Institut Pasteur, CRT, Paris, France; Faculty of Medicine, University of Kelaniya, SRI LANKA

## Introduction

Snake bite envenoming (SBE) is a major public health problem in many tropical countries in the developing world [1]. Conventional antivenom (AV) remains the main therapy and has been shown to reduce mortality in observational studies in several countries within sub-Saharan Africa (SSA) [2].While it is relatively available in other endemic settings in Asia and Latin America, for many years there have been major challenges with reliable supply of effective products within SSA. In 2016, the cessation of production of an excellent AV used in the region was announced, serving to galvanize the global health community into action towards the control of SBE, especially in the developing world. Since then, the World Health Organization (WHO) has recognized SBE as a category “A” neglected tropical disease (NTD) in 2017, adopted resolutions towards its control in 2018, and, in 2019, launched an ambitious “roadmap” towards control with the set targets of halving burden by 2030 [3,4].

In this Policy Appraisal, the challenges of AV supply are explored using the parameters of “security of supply,” a concept traditionally applied to food, energy, military, weapons, and, recently, medicines (e.g., insulin supply) [5,6]. The potential impact of expanding AV availability and supply in SSA is also be explored, building on our previous findings and cognizant of WHO Roadmap targets [4,7,8,9,10]. The financial resources needed to sustain AV supplies to achieve WHO Roadmap goals is estimated. Recommendations are offered towards attaining the targets.

## Security of supply

In SSA, where the burden is second only to that of Asia, there is only one AV producer based in South Africa [1,11,12]. Currently, several producers based in Asia, Europe, and Latin America produce varying amounts of AVs for use in SSA [11,12]. Almost all operate a business commercial model, with only few within the public model domain (e.g., the Instituto Clodomiro Picado [ICP], University of Costa Rica. In the past, production of several AVs were stopped largely due to poor affordability and dwindling demand and market share [13,14]. However, dependence on AV imports places African countries at supply risks (Table 1). These countries expend foreign currency on AV on the basis of burden and available resources. For instance, Burkina Faso spent an annual average of US$107,811 (or US$5,458 per million population) from 2010 to 2014, while the Nigerian government released only about US$192,000 (or US$980 per million population) for its snake bite program in 2017. This was only enough to treat about 4% of patients [14,15,16]. It has been argued that AV price should be around £3 for it to be affordable and allow for widespread accessibility [17]. Indeed, a public provider reduced price to as low as US$3.4 for a period in Burkina Faso in 2015 [16], while the Nigerian federal government and some state governments in Nigeria also provide it free. Nonetheless, resources are generally scarce to sustain AV availability in the long term, and rural facilities in the most vulnerable locations are usually not provided with sufficient AV supplies [15]. Current deployment approach of providing AV in urban tertiary centers fails to reach remote vulnerable populations. This is made worse in some endemic areas in certain countries such as parts of northeastern Nigeria, where “Boko Haram” insurgency has been ongoing posing additional challenges for access and delivery.

**Table 1:** Evaluating AV “security of supply” in SSA [5,6].

Security of Supply Criteria	Status and Comments
Diversity of Global Supply	There are 46 producers with 15 private manufacturing larger quantities. Supply in SSA is solely from external sources with exception of prohibitive South African products [12]
Production Ownership	African AV supplies originate from producers in India, Costa Rica, Egypt, Mexico, South Africa, Spain, and the United Kingdom. Currently, there are 12 poly-specific and 4 monospecific AVs in African markets [11]
Expenditure on AV	High cost affects health budgets and individual out-of-pocket expenditure among poor rural dwellers
Infrastructure	In many countries, procurement, supply chain management, and distribution network processes are weak
Sociopolitical stability of Countries	Exporting countries reasonably stable. Some importing countries are unstable (e.g., Central African Republic, DR Congo, South Sudan)
Economic Status of Countries	Most countries cannot support antivenoms’ expenditure, resulting in or suggesting the need for a regional solution
Stability of Prices	Prices of AV vary between countries, but they are generally high.
Affordability	Affordability is a challenge for patients and health systems. AV is purchased using domestic revenues not donor funds
Access and Equity	Grossly suboptimal and often related to affordability; endemic rural areas are grossly underserved. Deployment is through a “pull” rather than “push” system
Safety, Efficacy and Reliability of Supply	Many are liquid formulations requiring refrigeration. Some earlier AV formulations had unacceptable efficacy, safety and should be removed from the market by manufacturers or improved. WHO to monitor and standardize products vis GMP, prequalification, etc.
Vulnerability to Disruption	The fewer the producers, the more vulnerable the supply chain. Cessation of Fav-Afrique AV led to adverse incidences in CAR, Chad, Ghana, and Nigeria
Capacity to Adapt to Market Changes	Countries have little capacity to adapt given relatively few suppliers and resource constraints
Intellectual Property	This may be an issue with newer products in the pipeline

Abbreviations: GMP, good manufacturing practice

While there are 46 global producers of AV, currently, there are only 12 poly-specific and 4 monospecific AVs in the African market [11] (Table 1). In contrast to medicines, these products have all been developed using different snake venoms (toxins) and may have differing specificities, thereby precluding their use interchangeably. Only few of these AVs have undergone robust preclinical and clinical testing [18,19]. Many products were not properly assessed prior to importation and had suboptimal quality. For instance, AVs used following the cessation or stockout of Fav-Afrique in Chad, Central Africa Republic (CAR), and Ghana were associated with unacceptable rise in mortality [20,21,22].

Furthermore, the health systems in many countries are weak, with a lack of capacity to conduct quality assessments, and Health Care Workers (HCWs) are not fully trained to rationally use AVs. Overall, there is a need for strengthening logistics of procurement, distribution, and monitoring, as well as forecasting and rational utilization of AV. An analysis of AV “security of supply” is provided in Table 1.

## Potential public health impact and costs of providing antivenom and care

A recent estimate of SBE burden comprising years of life lost (YLL) due to premature deaths and years of life lived (YLD) with disability from amputation and posttraumatic stress disorder (PTSD) yielded 1.03 million disability-adjusted life years (DALYs) annually in SSA [7,10]. The WHO Roadmap targets halving snakebites and the resulting deaths and disabilities by the year 2030 [4]. Given that effective AV therapy following SBE compared to no AV therapy yielded an incremental benefit of an average 2.32 DALYs averted at an incremental cost of US$237 derived from our previous works (Table 2) [8,9], this leads to estimates of approximately 279,485 AV therapies needed at a cost of US$51.43 to 66.24 million annually for AV therapies priced at US$100 to 153 to achieve the targets by 2030 (Table 2). The needed amount equals to about US$47,690.74 to 61,427.50 per million population, respectively [23]. Thus, compared to current expenditure mentioned above, Burkina Faso will need to increase their spending by 8.74- to 11.25-fold, whereas Nigeria will need to increase spending by 48.66- to 62.68-fold, respectively, to attain the required funding to reduce the burden by 50%. Before then, reducing the burden by a one-quarter (or 25%) will mean averting 138,609 DALYs at a total base-case cost of $32,850,199. This lower target can be considered in the initial phases of the control efforts in the early 2020s.With political will and sustained advocacy, these expenditures may be achievable in few countries for a while, but, realistically, it will be difficult to get the whole continent to free fiscal space to sustain these allocations annually in the long term.

**Table 2:** Estimates of annual costs of halving the SBE burden in SSA to attain WHO Roadmap targets.

Item	Parameter	Number or Cost [$]	Reference/Assumption
A	Total DALYs incurred annually in SSA	1,029,209	Halilu et al 2019 [10]
B	50% of DALYs (WHO Roadmap targets)	514,605	WHO Roadmap, 2019 [4]
C	Impact of one AV therapy [in DALYs averted by 1 therapy]	average 2.32 DALYs^1^	Hamza et al 2016 [9]
D	AV treatments needed to achieve 50% reduction [b/2.32]	221,813	Hamza et al 2016 [9]
E	20% of patients will require at least a second AV dose before success [of d]	44,363	Abubakar et al 2010 [19]
F	AV wastage estimated at 5% [of d+e]	13,309	Usuf et al 2018 [26]
G	Total Annual AV needed to Achieve Roadmap target [d+e+f]	279,485	
H	Total cost at AV therapy cost of [$153^2^ + $84^3^ = $237 per Rx] (base-case)	66,237,945	Hamza et al 2016 [9]
I	Total cost at AV therapy cost of [$125^2^ + $84^3^ = $209 per Rx]	58,412,365	AV priced at $125; Brown 2012;Habib et al 2015 [8;13]
J	Total cost at AV therapy cost of [$100^2^ + $84^3^ = $184 per Rx]	51,425,240	AV priced at $100
K	Total cost at AV therapy cost of [$50^2^ + $84^3^ = $134 per Rx]	37,450,990	AV priced at $50
L	Total cost at AV therapy cost of [$10^2^ + $84^3^ = $94 per Rx]	26,271,590	AV priced at $10
M	Total cost at AV therapy cost of [$4^2^ + $84^3^ = $88 per Rx]	24,594,680	AV priced at $4; Theakston &Warrell, 2000 [17]
N	Total cost at AV therapy cost of [$315^2^ + $84^3^ = $399 per Rx]	111,514,515	AV priced at $315 produced in South Africa; Harrison et al 2017 [27]

^1^2.32 is derived as rural population weighted average from 16 West and Central African countries with values ranging from 0.84 to 2.87 DALYs averted per antivenom treatment [World Bank:^23^; ^28^]. The arithmetic mean equals 2.05

^2^Various AV prices are assumed $153 (base-case), then $125, $100, $50, $10, $4, and $315 the price of the antivenom produced by the South African Vaccine Producers (SAVP)

^3^$84 includes individual patient costs towards transportation and feeding and health facility costs towards basic laboratory tests, supportive care, managing adverse reactions, and transportation, storage, and refrigeration of antivenom.

As the public health impact of the AV is entirely driven by the reduction in YLL in our models [8,9], not YLD, there would be more survivors following AV use with actually more disabilities: amputation, posttraumatic stress disorder (PTSD), etc. This is because AVs have not been shown to improve complications such as necrosis, amputation or PTSD [1,24], and the probability of amputation was held constant at 3% and that probability will be applied to a greater number of survivors [8,9]. However, the reduction in YLL more than makes up for that. Ironically, targets for halving disabilities may be more difficult to achieve as more survivors will be left with complications not amenable to AV therapy.

The high costs of AV largely results from the cost of maintaining serpentarium, venoms, animal (horse) breeding, immunization (15 to 18 months), purification of IgG, digestion of IgG, quality control tests (mice tests) that must be carried out at each stage of production, and, for each batch produced, bottling, sometimes freeze-drying, distribution, and clinical studies. But above all, there is the risk of poor sales of a product that expires very quickly (especially with liquid formulation) further perpetuating price hikes [25]. Poor sales and utilization largely results from inaccessibility where it is needed, poor distribution network, lack of community awareness, poverty with poor affordability, lack of capacity of HCWs, and ineffective AVs with consequent erosion of confidence in orthodox medicines, resulting in most patients receiving care from traditional practitioners [15].

**Fig 1:**
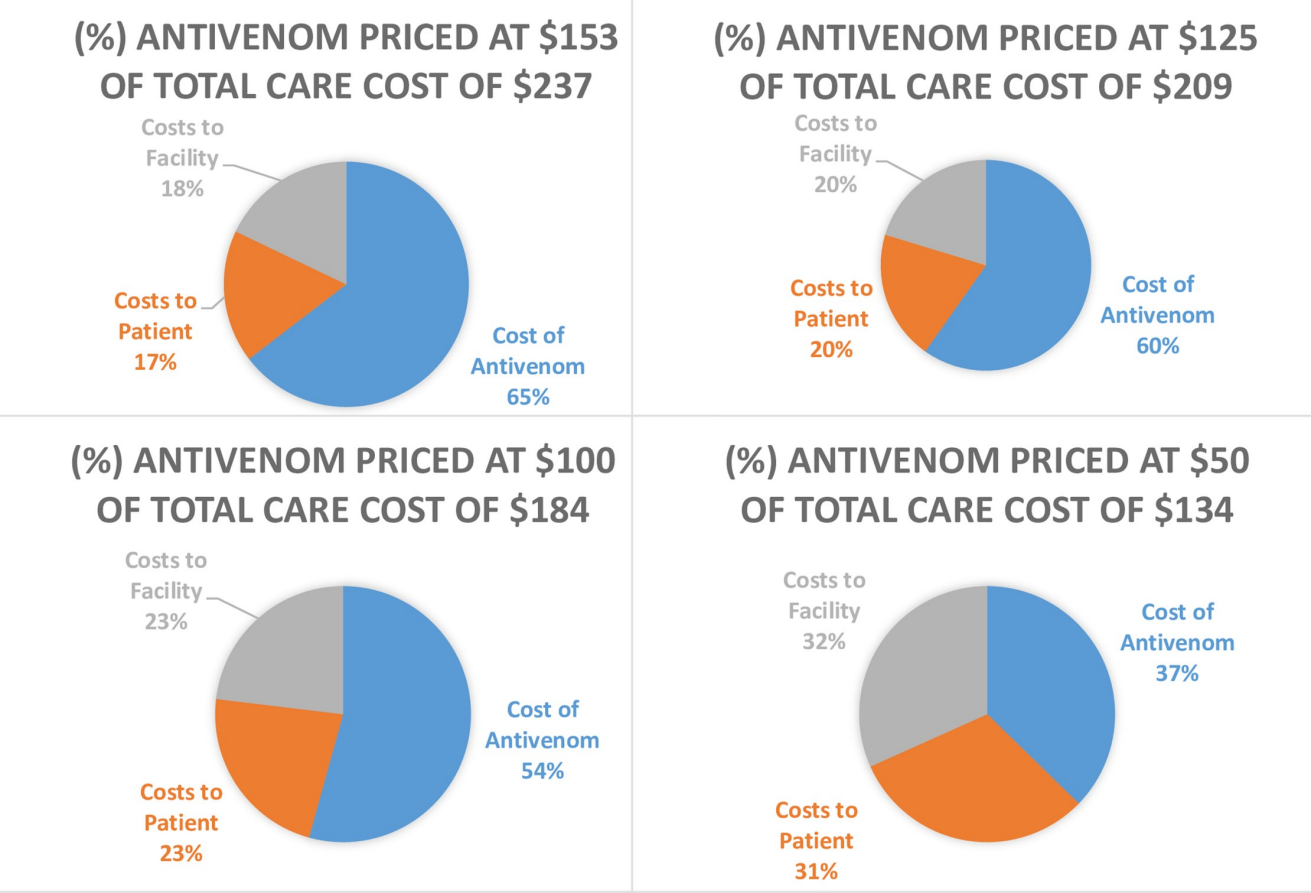
Contribution to costs of snake bite care based on varied antivenom prices.

It should be noted that actual AV cost accounts for 64.5% of the total in the base-case (Table 2). In scenarios with decreasing antivenom prices, the proportion spent towards AV similarly decreases to 59.8% at AV price of $125, 54.4% at $100, 37.3% at $50, 10.6% at $10, and 4.6% at $4. Therefore, the rest of the money will be needed towards providing for healthcare services and will be incurred either by the individual patient and or the state (Fig 1; Table 2).

In addition, substantial investments are needed to improve health capacity to deliver AV in the form of health infrastructure and health worker training. However, this latter investment can be leveraged on and integrated into other programs such as on immunization, NTDs, and other public health interventions. Thus, some of these investments should not be seen to be wholly for SBE/AV programs.

If SSA base-case context were applied to WHO Roadmap global targets using parameters presented in Table 2, AV therapy doses of 50,000 by year 2020, 500,000 by 2024, and 3 million by 2030 will annually avert 87,000 DALYs, 870,000 DALYs, and 5,220,000 DALYs by the respective times. This will be achieved at costs of $11.85 million, $118.5 million, and $711 million, respectively. Overall, our analysis yielded conservative estimates, as relatively lower number of SB cases, deaths, minimal AV wastage and higher weighted DALYs were used as inputs [9,10].

## Challenges and lessons towards sustaining the program

The WHO Roadmap is an ambitious useful starting point, and the targets are achievable, albeit at a huge cost. Currently, the resources are not available, and mechanisms for sustaining AV provisioning or stockpiling for SSA have to be developed. However, stockpiling is a major control effort for infections wherein vaccines against outbreaks and epidemics or drugs can be used (e.g., for influenza) or for mass drug administration against parasitic infections [29].The assumptions in these instances include an infection being transient with herd immunity developing following mass vaccination, thus negating subsequent annual use. Alternatively, that it is an emerging and epidemic condition necessitating containment, such as with oral cholera vaccine revolving stockpile, or a transient public health emergency of international concern, for which urgent containment is necessary and/or substantial subsidy is obtained for the product. Notable examples include Merck’s Mectizan Donation Program for river blindness; Bill & Melinda Gates Foundation (BMGF) support for Meningitis Vaccine Project to the meningitis belt countries; Global Drug Facility (GDF) for tuberculosis medicines; and Global Alliance for Vaccines & Immunizations (GAVI’s) support for routine childhood immunization in low and middle income countries (LMICs). As an NTD, global funding has been suboptimal for SBE, particularly when compared to other communicable NTDs despite its substantially higher burden [7]. Furthermore, as a noncommunicable disease (with no development of herd immunity) that recurs annually at more than 270,000 envenomings in SSA [1,10], there is the need for annual provision of large stock of AV therapies to be acquired and sustained.

## What is the way forward?

The WHO Roadmap is a laudable landmark towards controlling SBE in developing countries. It has 4 major complementary interventions: empowering and engaging communities; partnership, coordination, and resource generation; strengthening health systems; and provision of safe and effective treatments or AVs. According to the Roadmap, the last intervention will require the largest share or $49.73 million (36.6%) of the required $135.85 million to facilitate attainment of the antivenom targets from 2019 to 2030 [4].

## Improving efficiencies of existing AV programs

Given the considerable amount required to provide AV, all strategies to improve the funding resource base now and in the future should be pursued. However, humanitarian aid and international development assistance alone cannot support the WHO Roadmap and ensure sustainable financing. It is crucial that SSA countries contribute to the management of SBE through regional, governmental, and local resources. WHO should lead and coordinate a framework generating and revolving resources towards subsidized effective measures for controlling SB. The framework should include regional and international institutions, such as the African Union, WHO Regional Office for Africa (AFRO-WHO), World Bank, African Development Bank, and subregional blocks (e.g., Economic Community of West African States [ECOWAS], West African Health Organization [WAHO], and similar entities). Crucially, affected countries in the region should substantially increase their budgetary expenditure on SBE interventions and procurement of AV as well as incorporate its supply into the universal healthcare coverage (UHC) packages. Potentially through either individual country (governmental or nongovernmental) procurements or through a common joint (sub) regional funds, large scale pooled procurement can be affected. Such stockpiles will probably lead to reduction in prices through economies of scale. The latter mechanism can be tasked into resilient product market-shaping while ensuring provision of standardized, quality-assured, and affordable AVs based on WHO prequalification process. Such pooled procurement can be facilitated through development of newer more universal AVs effective against broader range of snakes in multiple countries in the region, a truly pan-African antivenom which will ensure and assure demand and facilitate mass production and price reduction through economies of scale.

To improve accessibility, a “hub-and-spoke” distribution and utilization network model should be implemented wherein rural facilities serve as satellites or spokes and are linked to major hospitals in urban hubs for referrals, linkages, support, and AV supplies. This way products and services are cascaded down from the center to the remote peripheral areas. A “push” model of supplying enough AVs to facilities for use when needed should be adopted as opposed to a “pull” model of trying to source for antivenom when the demand arises. Whenever feasible, products with longer shelf life (e.g., freeze dried antivenoms) should be encouraged for SSA. Given importation of products of unreliable quality, there is need to generate capacities in national regulatory agencies to be able to conduct preclinical and clinical tests to certify the quality of the antivenoms imported to their countries. Similarly, pregraduation training, in-service training of all cadres of HCWs on SB and AV use as well as improving or providing basic set-up for SB management should be implemented. There should be concurrent community health education highlighting preventive measures and the need for prompt hospital presentation for care and AV use following bite as well as continued engagement with health policy makers. Hopefully, this will improve demand and AV utilization, while facilitating budgetary allocation for its supply.

Although the AVs currently produced in SSA by the SAVP have been very effective in other subregions within the continent (e.g., West Africa), their production volumes have remained low, prohibitively expensive, and, generally, scarce to obtain. At its current price of $315 per vial [27], about $111.52 million will be required annually to attain the Roadmap targets (Table 2). At that price, AV will account for 79% of the total amount spent on care. All attempts should be made to substantially increase its production volume, widen distribution, and reduce prices.

At the same time, the number of intermediaries or “middle men” in the purchase chain should be reduced for both existing and planned locally produced AVs to curtail price hikes. However, care should be exercised to maintain, preserve, and revamp peripheral distribution chains and networks in order to improve access in rural areas.

## Exploring local antivenom production

Already, the WHO Roadmap encourages regional diversification and emergence of new public–private partnerships for antivenom production in LMICs, hoping it will contribute towards reaching the target of a 25% increase in the number of manufacturers by 2030 [4]. However, given the challenges outlined, development of local AV production within SSA should be seriously considered and promoted to improve the realistic likelihood of attaining the targets. To improve sustainability and viability, countries with similar snake fauna causing SBE should be encouraged to develop a manufacturing facility to supply their subregion, noting about £8–10 million upfront investment will be needed [30]. However, in addition to the initial capital investments, resources have to be allowed for the maintenance and sustenance of such production facilities which may not be insubstantial, especially in the early phases. But the potential local value chain addition that may result from establishing local manufacture should also be factored, e.g., employment and job creation. Additional funds to maintain and regularly procure the manufactured antivenom will still be required. To achieve the Roadmap’s targets from computations mentioned above with current populations [23] and with AV therapy priced at $100 to 153, Burkina Faso and Nigeria will need to provide approximately $942,000 to $1,214,000 annually and $9,342,000 to $12,032,000 annually, respectively, for antivenom procurements. Thus, the total investment for 1 to 2 production facilities still will be less than the annual amount SSA requires to purchase AVs from external sources using meager foreign reserves. Therefore, it would appear more prudent to initially plan maximum of about 2 such facilities for profitability and sustainability within the continent. Existing north–south and south–south collaborations on SB should be leveraged upon towards local production. An example is Nigeria’s EchiTAb project, wherein the government invested about £2 million towards development of antivenoms (EchiTAb G and EchiTAb Plus) using venoms from the country’s snakes with eventual plans for local production that never took place. In a similar arrangement, some manufacturers already produce AV for a number of countries in Latin America. Similarly, in Sri Lanka, where SB treatment costs the country about $10 million annually, with approximately two-thirds of the amount used to procure AV [31], a production facility is planned with arrangements for an initial AV development, provision, and agreement for subsequent production locally within Sri Lanka. Similar arrangements should be developed for SSA with willing global suppliers of affordable products with capacity for surplus production and plans for eventual local manufacture. Several modalities towards this initiative should be considered although public to private partnerships would appear to be most logical and sustainable. Other modalities such as public to public partnerships, as has been done in Latin America, and/or private-private partnerships should all be explored.

In addition to the financial investments needed for local AV production, there is the technical complexity of establishing and sustaining production locally. Therefore, other pragmatic approaches should be considered, such as implementing a gradual strategy or step-by-step approach as recently suggested by these authors [32]. This may entail an initial development of serpentarium to collect high quality venoms that could be provided to regulatory agencies for quality control and to manufacturers. This could be followed by a stage in which countries develop farms for keeping and immunizing horses in order to collect hyperimmune blood and plasma to be sent to manufacturing laboratories for AV production and, finally, the stage of full implementation of production in some African countries. This sequential approach with gradual consolidation of local capacities can be established through an international framework with active engagement of WHO.

In considering local AV production, it should be decided whether a pan-African or regional AV is developed. In favor of a continental polyvalent AV may be a larger market and easier marketability with only one (or few) products, especially as geographic distribution of venomous species does not follow national borders though regulation and marketing does. However, conceptual issues due to the evolution of taxonomy, such as the recent cleavage of *Naja melanoleuca* into 4 or 5 species, may pose more challenges and warrant consideration of a regionally more restricted AV. Ensuring AVs with paraspecificity in experimental and preclinical testing across wide ranging species might serve as a mitigation step. These initiatives should be coordinated and guided by the governments in the region.

## Facilitating next generation therapies

Finally, availability of monoclonal antibody therapies has been limited to wealthier countries around the world due to their high manufacturing costs, but they are safer and more efficacious. Making them more readily affordable and globally accessible, as next generation improved snake bite therapies, will hasten achievement of WHO Roadmap targets. As AV and the next generation therapies are immunobiologicals, GAVI and philanthropic foundations should be approached towards subsiding them with an initial investment followed by a self-sustaining revolving fund model for SSA countries. Counterpart funding should also be provided by the affected countries in order to access subsidized products. To champion these strategies, civil society has to be galvanized to emerge and proactively facilitate these processes.

## Conclusion

In summary, SBE is a major public health concern that causes substantial morbidity and mortality in SSA. Despite highly favorable antivenom cost-effectiveness and an annual burden estimated at over 1 million DALYs, resource allocation and consequent antivenom supply, access, affordability, and availability have been grossly inadequate. The recently launched Roadmap strategy for prevention and control of SBE by WHO seeks to halve the burden by 2030. It utilizes multifaceted approaches involving phased-in antivenom stockpiling, hoping to make available 3 million treatments annually at full roll-out by 2030. However, in SSA, an estimated annual amount of $51 to 66 million will be needed to halve the burden based on AV therapy prices of $100 to 153, with 54 to 65% of the amount required for AV procurement. Furthermore. as a noncommunicable disease, antivenom stockpiling for SBE may pose peculiar challenges to sustain in the long term. Innovative approaches such as subregional joint pooled AV procurements, reduced acquisition costs through volume procurements and economies of scale, and inclusion of AV supply in subsidy-oriented sustainable revolving programs facilitated by international and regional health agencies are recommended. It is imperative for countries to increase budgetary allocation as well as explore establishing local production facilities through public and private partnerships using either north–south or south–south collaborations. Logistics of procurement, distribution, and monitoring as well as forecasting and rational utilization of AV should be strengthened in SSA.
